# Validation of the clinical assessment scale for autoimmune encephalitis in a severe autoimmune encephalitis cohort

**DOI:** 10.3389/fimmu.2024.1490804

**Published:** 2024-12-02

**Authors:** Yu He, Fangfang Li, Ali Yang, Chen Yu, Yifan Wang, Jing Zhao, Weizhou Zang

**Affiliations:** ^1^ Department of Neurology, Henan University People’s Hospital, Zhengzhou, China; ^2^ Department of Neurology, Henan Provincial People’s Hospital, Zhengzhou, China; ^3^ Department of Neurology, Zhengzhou University People’s Hospital, Zhengzhou, China; ^4^ Department of Medical Imaging, Zhengzhou University People’s Hospital, Zhengzhou, China

**Keywords:** severe autoimmune encephalitis, CASE, mRS, validation, prognosis

## Abstract

**Objective:**

The Clinical Assessment Scale for Autoimmune Encephalitis (CASE) is a novel tool tailored specifically for evaluating the severity of autoimmune encephalitis (AE). However, its application in severe AE patients is limited. This study aimed to evaluate the reliability and validity of the CASE and explore its clinical significance in a severe AE cohort.

**Methods:**

The relevant clinical characteristics, laboratory data, and prognosis of patients diagnosed with severe AE between April 2017 and April 2023 were collected. The CASE and modified Rankin scale (mRS) were performed at admission, discharge, and 1-year follow-up, respectively. The reliability of CASE was validated by calculating the Cronbach’s alpha value. The validity was evaluated by calculating the Spearman’s rank correlation with the corresponding mRS. Univariate and multivariate logistic regression were utilized to identify risk factors for poor prognosis.

**Results:**

A total of 140 patients were recruited for the study. The CASE scale presented great internal consistency, with Cronbach’s α value of 0.768 for the total score. The Spearman’s rank correlation analysis revealed strong criterion validity between CASE and mRS, with coefficients of 0.68, 0.92, and 0.95 at admission, discharge, and 1-year follow-up, respectively (all *p* < 0.001). ROC analysis identified CASE score at admission served as a promising predictive marker for clinical response to treatment, with an AUC of 0.67 (95% CI: 0.57-0.77, *p* = 0.003). The optimal cut-off point was 22.5. At 1-year follow-up, 72/140 (51.4%) patients achieved good functional status (mRS, 0-2). Multivariate logistic regression confirmed that higher CASE scores on admission and older age at onset were associated with poor short-term as well as 1-year prognosis, respectively. In addition, no clinical response to treatment (OR = 40.499; 95% CI: 7.077-231.746, *p* < 0.001) and longer duration of hospitalization (OR = 1.071; 95% CI: 1.017-1.128, *p* = 0.010) were associated with poor function states at 1-year follow-up.

**Conclusion:**

The CASE has proven suitable for evaluating disease severity and prognosis in severe AE patients. Besides, CASE score, age at disease onset, hospital stays, and response to immunotherapy are identified as independent risk factors for unsatisfactory prognosis in severe AE patients.

## 1 Introduction

Autoimmune encephalitis (AE) is a group of rapidly progressive neuroinflammatory diseases linked to autoantibodies targeting neuronal surface, synaptic, or intracellular antigens. With an incidence of up to 12.6 per 100,000, AE stands as the second most frequent cause of encephalitis after infectious encephalitis (1–3). AE is a highly heterogeneous disease with distinct clinical features and varying severity. The main clinical characteristics of AE include seizures, psychiatric symptoms, cognitive disorders, language dysfunction, disturbance of consciousness, autonomic dysfunction, and movement disorders (4, 5). The symptoms of AE can range from the mildest memory loss to the most severe consistently progressive disorders of consciousness (3, 6–8). During the acute phase of AE, some patients develop a fatal condition due to rapid progression of immune-inflammatory response and often require long-term intensive care. Most patients with severe AE spend a lengthy time in the Intensive Care Unit (ICU), which is difficult and costly to treat and lays a huge strain on their families. Several studies have verified that approximately half of severe AE patients have unsatisfactory outcomes (6, 9, 10). Therefore, identifying high-risk patients with poor prognoses and adopting more aggressive treatment is essential (11–13).

Owing to the absence of specialized tools, the modified Rankin Scale (mRS) is commonly employed to evaluate disease severity and outcomes in AE patients (14). Nonetheless, this scale is focused primarily on motor ability, while AE patients frequently appear with kinds of non-motor symptoms, such as psychosis, seizures, decreased level of consciousness, and language problems. Using mRS to appraise the highly heterogeneous clinical manifestations of patients with AE is improper. Moreover, patients with severe AE are frequently given 4-5 points using mRS, which does not allow for better distinction and precise evaluation of the disease severity (6). CASE was specifically devised in 2019 to evaluate the intensity of AE, and it has nine items that cover the frequent clinical symptoms of AE (15). Compensating for the shortcomings of traditional assessment tools in evaluating non-motor manifestations of AE, CASE provides a more detailed and specific assessment of disease severity by quantifying multiple symptoms of patients.

Several studies have proven the validity and reliability of CASE in different AE cohorts (16–18). However, none specifically targeted the evaluation of the performance of CASE in severe AE patients. This study endeavors to assess the performance of CASE and explore the factors that may increase the risk of a poor outcome for patients with severe AE.

## 2 Materials and methods

### 2.1 Participants

The study encompassed patients with severe AE who were admitted to Henan Provincial People’s Hospital during the period from April 2017 to April 2023. The following were the inclusion criteria: (1) Fulfilled the diagnostic standards established by Graus et al. in 2016 (8). (2) Severe neurological damage at disease onset, with mRS score of 4–5. (3) Due to symptoms such as status epilepticus, decreased consciousness, or respiratory failure, the Intensive Care Unit (ICU) admission was necessary for better treatment. The exclusion criteria were: (1) mRS score of 0–3. (2) the presence of a concurrent systemic autoimmune disorder at the onset of the illness. (3) Incomplete records.

### 2.2 Data collection

Data collection included baseline demographics, antibody test results, clinical manifestations, laboratory indicators, neuroimaging findings, and treatment plans. Following the guidelines for AE, the current severe AE cohort received high-dose glucocorticoids, immunoglobulin, or plasma exchange as first-line treatment, and rituximab, cyclophosphamide, tocilizumab, and additional immunotherapy served as second-line treatment (19–21). For patients with refractory severe AE who have not shown significant improvement despite 1-2 months of second-line treatment, intensification of immunotherapy may be contemplated following rigorous screening procedures, such as adding intravenous tocilizumab (22–24). Abnormal MRI presentations in patients with AE include T2 and FLAIR hyperintense lesions, etc. Lumbar punctures were performed before treatment. In addition, we calculated the Qalb (CSF albumin/Serum albumin) and NLR (Neutrophil-to-lymphocyte ratio). The Qalb is a valuable indicator of blood-brain barrier dysfunction. The NLR is an inflammatory biomarker that is responsive to systemic inflammation. In recent years, Qalb and NLR have gradually been discovered to be strongly connected with the severity and bad outcomes of AE patients (13, 25, 26). All data were collected before treatment.

### 2.3 Antibody test

Samples of CSF and blood were sent to a single laboratory for antibody tests. The laboratory conducted tests using indirect immunofluorescence (IIF) or cell-based assay (CBA) to identify the types and titers of antibodies according to the supplier’s instructions (27, 28). For each patient, at least six primary antibody types were detected: anti-N-methyl-D-aspartate receptor (NMDAR) antibody, anti-contactin-associated protein-like 2 (CASPR2) antibody, anti-α-amino-3-hydroxy-5-methyl-4-isoxazole propionic acid 1 receptor (AMPA1R) antibody, AMPA2R antibody, anti-gamma-aminobutyric acid-B receptor (GABA_B_R) antibody, and anti-leucine-rich glioma-inactivated 1 (LGI1) antibody. Additionally, there were optional antibody types such as anti-dipeptidyl-peptidase-like protein-6 (DPPX) antibody, anti-GABA_A_R antibody, anti-mGluR5 antibody, anti-myelin oligodendrocyte glycoprotein (MOG) antibody, anti-glutamic acid decarboxylase 65 (GAD65) antibody, anti-Ma2 antibody, anti-Hu antibody, and anti-Dopamine 2 receptor (D2R) antibody, among others (29, 30). Antibody titer levels were categorized as low (+, with a 1:10 dilution in blood or 1:1 in CSF), moderate (++, with a dilution of up to 1:100 in blood or up to 1:10 in CSF), or high (+++, with a dilution of 1:320 or higher in blood or 1:32 or higher in CSF). The initial dilutions used for comparison were 1:1 for CSF and 1:10 for serum.

### 2.4 Scale assessment

The mRS and CASE were conducted at three-time points: at admission, discharge, and 1-year after discharge. The CASE score spans from 0 to 27 points. It contains nine items, each evaluated from 0 to 3 (15). The scale of seizure included none (score = 0), controlled seizures (score = 1), refractory epilepsy (needed dose increase or add-on treatment with any antiepileptic drug, score = 2), and status epilepticus (score = 3). Brainstem dysfunction manifested as gaze paresis, requiring tube feeding for nutritional support, and ventilator assistance for respiratory management due to central hypoventilation. This item was scored based on the number of symptoms. The assessment of language and memory function was primarily based on communication and observation. The remaining items were classified as none (score = 0), mild (score = 1), moderate (score = 2), and severe (score = 3) according to the severity of symptoms. In comatose patients, assessment could be based on seizure, dyskinesia/dystonia, and brainstem dysfunction, while all other items were automatically assigned a score of 3 (18). The CASE score is calculated as the total of the individual item scores. We utilized the mRS score as a standard tool for severe AE to assess the validity of CASE.

Two neurologists, H-Y and L-FF, unaware of the research purposes, independently evaluated the scales through comprehensive medical record reviews. Any discrepancies were reconciled through meticulous discussions until a unanimous consensus was reached. In case of disagreement, a third senior neurologist, Z-WZ, would make the ultimate determination. Following discharge, clinical data was gathered via telephone, WeChat, or outpatient consultations with two neurologists, and CASE and mRS scores were re-evaluated at 1-year after discharge.

### 2.5 Prognosis and operational definitions

Clinical response to treatment was categorized as meaningful clinical improvement (mRS improvement ≥1 point) and no clinical improvement (mRS improvement <1 point) (12). Clinical relapse was characterized as the emergence of new symptoms or worsening of existing conditions, occurring at least 2 months after an initial improvement or stabilization. Good prognosis was indicated by mRS scores of 2 or less, whereas poor prognosis corresponded to scores greater than 2.

### 2.6 Statistical analysis

SPSS 26.0 (SPSS Inc, Chicago, IL) was utilized to analyze all collected data. Categorical variables were represented by frequencies and percentages, while non-normally distributed continuous variables were presented as medians and ranges. Categorical variables were analyzed by Pearson’s chi-squared test. Continuous variables with two subgroups were compared using the Mann–Whitney U test. Cronbach’s alpha was conducted to examine each item’s internal consistency, and a value > 0.70 was judged to have great reliability. Spearman’s correlation analyses were performed on the CASE score and mRS total score to assess criterion validity. Both univariate and multivariate logistic regression analyses were conducted to identify risk factors linked to poor outcomes. Variables with *P* < 0.05 in univariate tests proceeded to multivariate logistic regression.

Receiver operating characteristic (ROC) curves were employed to evaluate the predictive values of these hazardous factors in forecasting poor outcomes. Additionally, ROC curves were also employed to investigate the predictive efficacy of CASE score to clinical response. A *P*-value less than 0.05, on both sides, indicated statistical significance.

### 2.7 Ethics statement

The Ethics Committee of Henan Provincial People’s Hospital reviewed and approved the study [approval number (2022): Ethics Review No.129]. The study adhered strictly to both local legislative frameworks and institutional guidelines, with all data being gathered anonymously and stored in a database. Conforming to national laws and institutional standards, participants or their legal representatives were not mandated to provide written informed consent for their participation. Instead, during their initial visit, parents signed a consent form, agreeing that the clinical data of their children could be utilized for various purposes, including clinical research, epidemiology, pathological studies, training endeavors, and the enhancement of knowledge, care, and preventive measures.

## 3 Results

### 3.1 Patient characteristics

The clinical progressions of 140 severe patients with AE from Henan Provincial People’s Hospital were investigated. Among these patients, the median age was 44 years, ranging from 3 to 87 years. The female-to-male ratio was 3:5. The median length of hospital stays was 23 days, ranging from 6 to 123 days. 25 (17.9%) patients tested positive for anti-NMDAR antibodies (15 females and 10 males), 13 (9.3%) for other antibody types (3 females and 10 males), and 102 (72.9%) were antibody-negative. The other types included CASPR2 (1 female and 5 males), GABA_B_R (1 female and 2 males), LGL1 (1 female), LgLON5 (1 male), mGluR5 (1 male), and MOG (1 male) antibodies. The median mRS score at admission was 5 (mRS 4: n=30; 5: n=110).

The more frequent clinical presentations were memory impairment, psychiatric disturbances, decline of consciousness, and dyskinesia. Nearly all patients exhibited these symptoms, albeit with varying degrees of severity. A total of 88(62.8%) patients presented with epileptic symptoms, including 2 with controlled epilepsy, 40 with refractory epilepsy, and 46 with status epilepticus. In our cohort, psychiatric symptoms primarily encompassed mood dysfunction, delusion, hallucination, disinhibition, aggression, among others. There were 10(7.14%) patients with mild mental disorders (not requiring medical intervention as they did not affect daily activities), 25(17.9%) patients with moderate mental disorders (requiring intervention due to interference with daily activities), and 105(75.0%) patients with severe mental disorders (needs continuous care or admission because of psychiatric symptom or unable to check). Regarding memory impairment, 2(1.43%) patients had mild impairment (not affecting daily activities), 33(23.6%) had moderate impairment (interfering with daily activities), and 105(75.0%) had severe impairment (no recent memory or unable to communicate). 60 patients (42.9%) were admitted to ICU due to central hypoventilation necessitating respiratory support. The baseline characteristics of the cohort are presented in **Table 1**. During follow-up, 20 (14.3%) patients died, and 43 (30.71%) patients suffered relapses. Furthermore, 38 (27.1%) patients had favorable short-term clinical outcomes, and 72 (51.4%) patients had good 1-year clinical outcomes (mRS score ≤2).

**Table 1:** Baseline data (N, median, %).

Characteristics		Total	Antibody positive		Antibody negative
			NMDAR	Other types	
Number		140	25(17.9)	13(9.3)	102(72.8)
Gender	Female	53(37.9)	15(60.0)	3(23.1)	35(34.3)
	Male	87(62.1)	10(40.0)	10(76.9)	67(65.7)
Age at onset(years), median(range)		44 (3–87)	26 (7–66)	60 (12–73)	49 (3–87)
Clinical features, n(%)	Seizures	88(62.8)	23(92.0)	11(84.6)	54(52.9)
	Memory dysfunction	138(98.5)	24(96.0)	12(92.3)	102(100.0)
	Psychiatric symptoms	140(100.0)	25(100.0)	13(100.0)	102(100.0)
	Consciousness	121(86.4)	22(88.0)	11(84.6)	88(86.3)
	Language problem	136(97.1)	24(96.0)	12(92.3)	100(98.0)
	Dyskinesia/dystonia	140(100.0)	25(100.0)	13(100.0)	102(100.0)
	Gait instability and ataxia	136(97.1)	25(100.0)	13(100.0)	98(96.1)
	Brain stem dysfunction	130(92.9)	23(92.0)	13(100.0)	94(92.2)
	Weakness	111(72.3)	18(72.0)	11(84.6)	82(80.4)
MRI	Normal	45(36.0)	8(32.0)	5(38.5)	32(31.4)
	Abnormal	80(64.0)	14(56.0)	6(46.2)	60(58.8)
Immunotherapy	IVGG	118(84.3)	25(100.0)	11(84.6)	82 (80.4)
	High dose glucocorticoid	140(100.00)	25(100.0)	13(100.0)	102(100.0)
	Plasma exchange	12(8.6)	4(16.0)	1(7.7)	7(6.9)
	Rituximab	6(4.3)	1(4.0)	1(7.7)	4(3.9)
	Others	32(22.9)	12(48.0)	3(23.1)	17(16.7)
Time from admission to initiation of immunotherapy(days)		5(0-23)	4 (0–17)	6 (2–21)	6 (0–23)
Hospital stays(days)		23 (6–123)	29 (8–123)	26 (11–67)	21 (6–87)
ICU stays(days)		8 (1–87)	9 (1–83)	6 (2–29)	8 (8–87)
Presence of antibodies, n (%)	CSF	34(24.3)	25(100.0)	9(69.2)	/
	Serum	22(15.7)	18(72.0)	4(30.8)	/
Laboratory index	WBC (10^9^/L, M, IQR)	9.38(2.37-43.60)	9.55(4.63-20.11)	8.13(2.37-18.54)	9.18(3.08-43.60)
	CRP (mg/L, M, IQR)	11.35(0.10-200.00)	2.61(0.50-103.89)	11.61(0.50-70.74)	16.79(0.10-200.00)
	CSF albumin (g/L, M, IQR)	0.42(0.11-2.54)	0.36(0.11-1.17)	0.38(0.13-0.95)	0.46(1.15-2.54)
	Serum albumin (g/L, M, IQR)	35.80(25.30-47.90)	38.40(31.00-47.30)	34.60(31.10-46.10)	35.65(25.30-47.90)
	Qalb (M, IQR)	12.06(3.19-70.75)	8.96(3.34-34.21)	11.38(3.19-29.14)	13.70(4.14-70.75)
	NLR (M, IQR)	6.10(0.60-100.20)	5.90(2.80-67.00)	6.10(0.60-45.70)	6.35(1.10-100.20)

mRS, the modified Rankin scale; CASE, The Clinical Assessment Scale for Autoimmune Encephalitis; ICU, Intensive Care Unit; WBC, white blood cell; CRP, C-reactive protein; NLR, neutrophil-to-lymphocyte ratio; Albs, Serum Albumin; Albcsf, CSF Albumin; Qalb, Albcsf-to-Albs ratio.

### 3.2 Reliability and validity of the CASE

To verify if the CASE items were suitable for assessing the severe AE cohort, we evaluated the CASE’s internal consistency. The Cronbach’s alpha coefficient for the overall CASE score was 0.768, and the Cronbach’s alpha values of individual CASE items contributing to the total score ranged from 0.222 to 0.590. The items with the lowest and highest Cronbach’s alpha coefficients were item 3 (Psychiatric symptoms) and item 9 (Weakness), respectively. The Spearman’s correlation coefficient between mRS and total CASE score was 0.679, indicating good criterion validity. The correlation coefficients between each item and mRS score ranged from 0.207 to 0.678. Furthermore, there was an excellent correlation between CASE and mRS scores at both discharge and follow-up (ρ = 0.92, and ρ = 0.95*, p* < 0.001) (**Table 2**).

**Table 2:** Spearman correlation analyses of CASE score and mRS total score.

CASE	mRS(Total score)	
	ρ	*P*
On admission		
Seizure	0.027	0.014
Memory dysfunction	0.450	< 0.001
Psychiatric symptoms	0.462	< 0.001
Consciousness	0.539	< 0.001
Language problem	0.393	< 0.001
Dyskinesia/dystonia	0.660	< 0.001
Gait instability and ataxia	0.678	< 0.001
Brainstem dysfunction (number of symptoms)	0.505	< 0.001
Weakness	0.656	< 0.001
Total score	0.679	< 0.001
At discharge		
Total score	0.920	< 0.001
At 1 year		
Total score	0.950	< 0.001

CASE, The Clinical Assessment Scale for Autoimmune Encephalitis; mRS, the modified Rankin scale.

**Figure 1:**
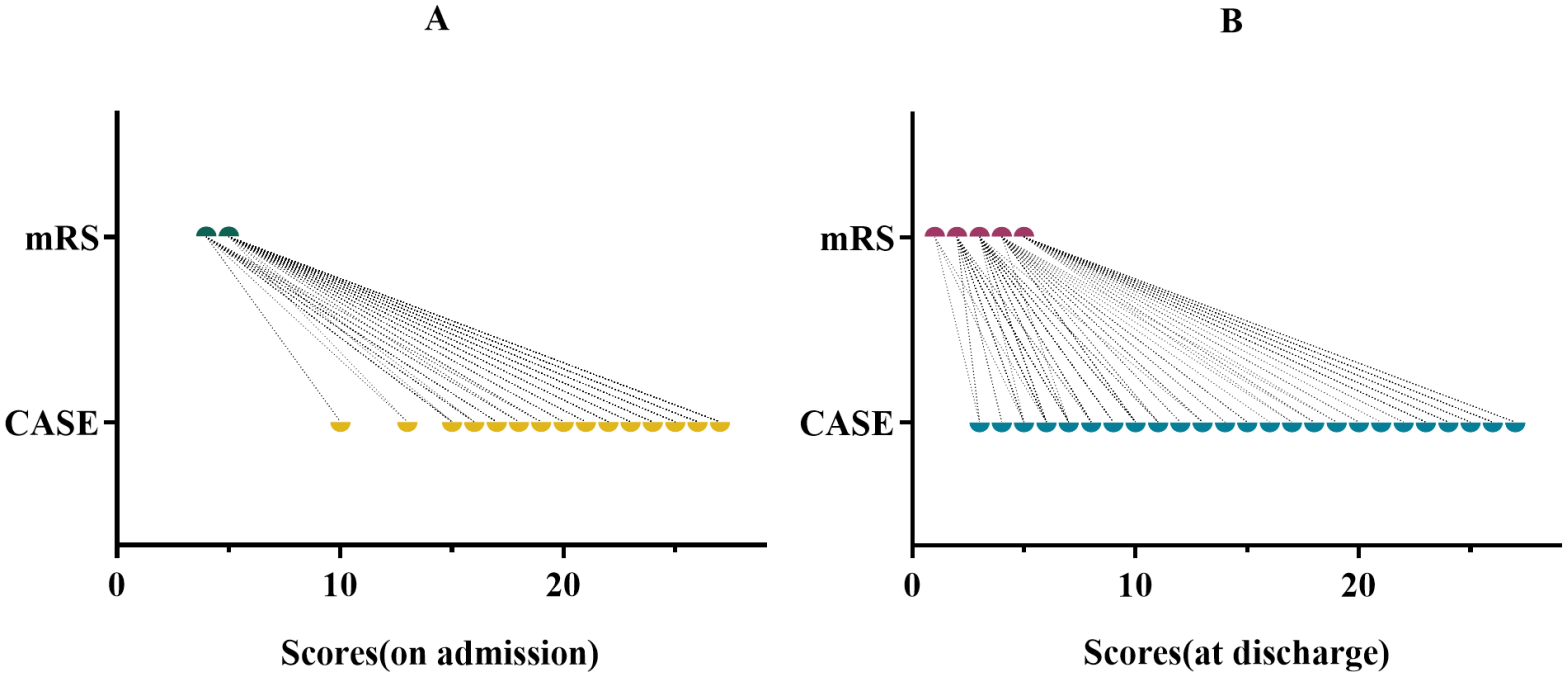
CASE and mRS scores for the same period in the same patient. **(A)** for admission. **(B)** for discharge.

In addition, mRS and CASE scores were collected at admission and discharge for each patient (**Figure 1**). We clearly found that the range of CASE score was wider than mRS score within the same range. This suggests that CASE is more accurate and sensitive than mRS in capturing disease severity and progression.

### 3.3 CASE score and treatment

**Figure 2:**
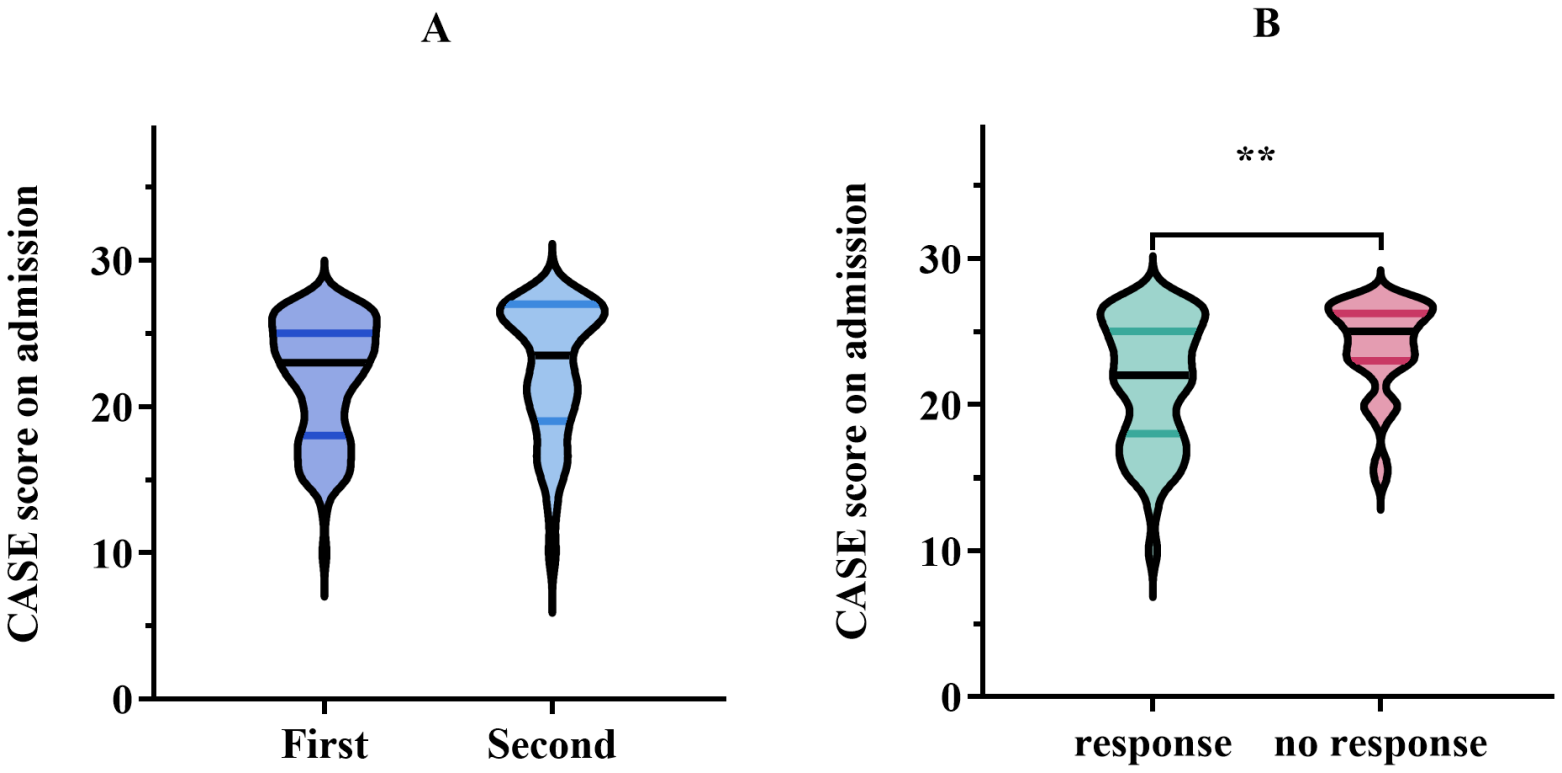
**(A)** Comparison of CASE score on admission using first-line treatment versus second-line treatment. Data are presented as median (interquartile range, IQR). **(B)** CASE score on admission in the response group (improvement in the mRS, ≥1 point) *vs*. no response group (improvement in the mRS, <1 point) after treatment. The meaning of the symbol ** indicated *P* < 0.01.

All patients underwent first-line therapy, which included glucocorticoids, IVIG, or a combination of both. Only 38 patients (27.1%) were administered second-line immunotherapy consisting of rituximab, cyclophosphamide, Ofatumumab, tocilizumab, or a combination of cyclophosphamide and tocilizumab. Of the 38 patients mentioned, the majority of patients (36 out of 38) were treated with a single second-line drug, whereas a small minority (2 out of 38) received a more complex treatment plan involving a combination of cyclophosphamide and tocilizumab. The median CASE scores for patients solely treated with first-line therapy and those who also experienced second-line therapy were 23 and 24, respectively. However, no notable difference in CASE scores was observed between the two therapeutic regimens (*p* = 0.272) (**Figure 2A**). Similarly, no significant variation in CASE scores was detected between antibody-positive and antibody-negative patients at the time of admission and discharge (*p* = 0.496; *p* = 0.065, respectively).

**Figure 3:**
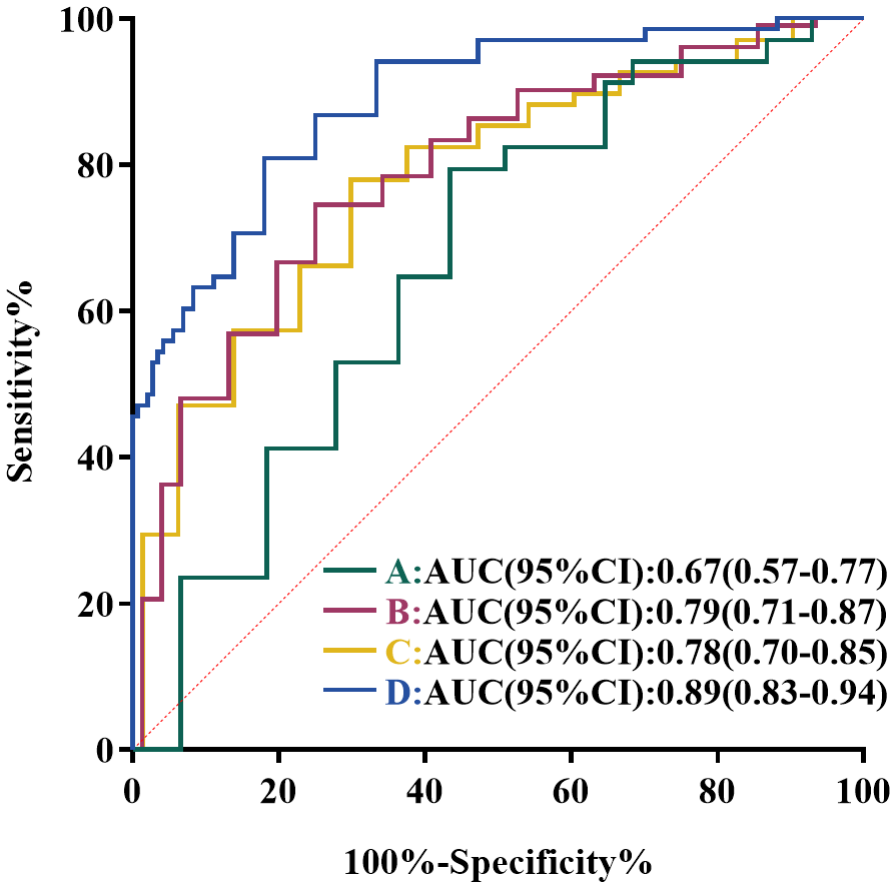
ROC curve analysis of the predictive value of CASE score in severe AE patients. **(A)** ROC Curve of CASE score on admission for predicting clinical response to immunotherapy. **(B)** ROC curves of CASE score on admission for predicting short-term poor prognosis. **(C)** ROC curves of CASE score on admission for predicting 1-year poor prognosis. **(D)** ROC curves of CASE score at discharge for predicting 1-year poor prognosis.

The median CASE score of patients with meaningful clinical improvement (mRS improvement ≥ 1 point) was 22, while the median CASE score of patients with no clinical improvement (mRS improvement < 1 point) was 25, showing a markedly different (*p* = 0.003) (**Figure 2B**). Next, the ROC curve analysis was employed to assess the predictive ability of the CASE score on admission for clinical response to treatment. As shown in **Figure 3A**, the CASE score had a superior predictive ability for meaningful clinical improvement to treatment in the severe AE cohort (AUC: 0.67, 95% CI: 0.57–0.77, *p* = 0.003), and a cutoff of 22.5 indicated that Patients with scores above this threshold were more likely to have no clinical improvement to treatment (sensitivity, 79.4%; specificity, 53.8%).

### 3.4 CASE score and length of hospital stays

The median length of hospital stays for severe AE patients was 23 (range: 6-123 days). And the median length of ICU stays was 8 (range: 1-87 days). The factors that were significantly positively linked with hospital stays were ICU stays (ρ _=_ 0.64, *p* < 0.001), mRS score at admission (ρ = 0.26, *p* = 0.002), and CASE score on admission (ρ = 0.40, *p* < 0.001). However, we did not find that age at onset (ρ _=_ -0.16, *p* = 0.06) and gender (ρ _=_ -0.01, *p* = 0.88) were relevant to the length of hospital stays.

### 3.5 CASE score and short-term prognosis

**Figure 4:**
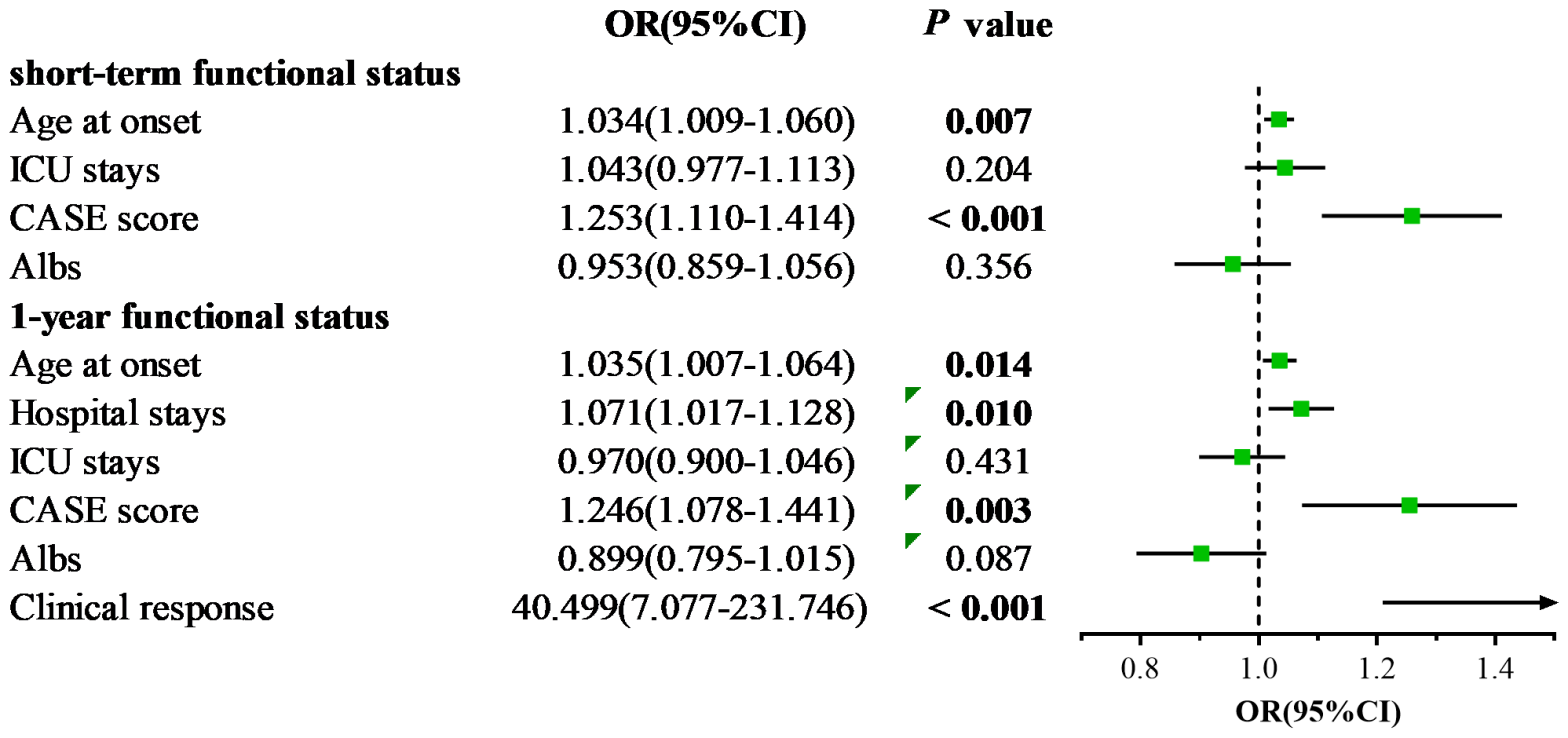
Forest plots of multivariable logistic regression analysis for factors linked to poor outcomes.

Primarily, we performed univariate logistic regression analysis to select factors linked to short-term prognosis. It showed that age at onset (*p* < 0.001), ICU stays (*p* = 0.006), CASE score on admission (*p* < 0.001), and serum albumin level (Albs) (*p* = 0.010) were relevant to bad short-term prognosis, as shown in **Table 3**. Then, we incorporated these variables into multivariable logistic regression analysis. The results revealed that older age at onset and higher CASE score on admission were conspicuously related to poor short-term prognosis (OR = 1.034; 95% CI: 1.009–1.060; *p* = 0.007 and OR = 1.253; 95% CI: 1.110–1.414; *p* < 0.001, respectively) (**Figure 4**).

**Table 3:** Univariate logistic regression analysis of factors related to poor prognosis.

Variables	Short term prognosis		1-year prognosis	
	OR (95%CI)	*P*	OR (95%CI)	*P*
Gender	1.225(0.574-2.626)	0.600	0.676(0.341-1.342)	0.236
Age at onset (years)	1.037(1.017-1.058)	**< 0.001**	1.032(1.014-1.050)	**< 0.001**
Time from admission to initiation of immunotherapy(days)	1.056(0.979-1.140)	0.161	1.063(0.997-1.132)	0.061
Hospital stays	–	–	1.042(1.017-1.068)	**0.001**
ICU stays	1.104(1.029-1.184)	**0.006**	1.085(1.033-1.139)	**0.001**
antibody	1.280(0.540-3.033)	0.575	1.674(0.788-3.554)	0.180
CASE score on admission	1.304(1.173-1.451)	**< 0.001**	1.311(1.178-1.459)	**< 0.001**
CASE score at discharge	–	–	1.344(1.211-1.492)	**< 0.001**
WBC	0.979(0.920-1.043)	0.511	1.000(0.943-1.061)	0.994
CRP	1.010(1.000-1.021)	0.060	1.011(1.003-1.019)	**0.010**
NLR	1.007(0.973-1.043)	0.677	1.026(0.990-1.063)	0.158
Albs	0.899(0.828-0.975)	**0.010**	0.885(0.820-0.954)	**0.002**
Albcsf	1.177(0.517-2.678)	0.697	0.714(0.347-1.471)	0.714
Qalb	1.011(0.981-1.042)	0.466	0.996(0.972-1.021)	0.760
Clinical response to treatment	–	–	31.111(7.054-137.222)	**< 0.001**
relapse	–	–	1.744(0.844-3.606)	0.133

OR, odds ratio; CI, confidence interval; WBC, white blood cell; CRP, C-reactive protein; NLR, neutrophil-to-lymphocyte ratio; Albs, Serum Albumin; Albcsf: CSF Albumin; Qalb: Albcsf-to-Albs ratio. The meaning of the bold values indicated statistical significance.

### 3.6 CASE score and 1-year prognosis

Next, we explored the performance of CASE in predicting 1-year prognosis in severe AE cohort. Univariate logistic regression analysis demonstrated that age at onset (*p* < 0.001), hospital stays (*p* = 0.001), ICU stays (*p* = 0.001), CASE score on admission (*p* < 0.001), CASE score at discharge (*p* < 0.001), CRP on admission (*p* = 0.010), Albs on admission (*p* = 0.002) and clinical response to treatment (*p* < 0.001) were relevant to poor 1-year functional status (**Table 3**). Multivariable logistic regression analysis then verified that several factors significantly increase the risk of poor 1-year prognosis: older age at disease onset, higher CASE score at admission, longer hospital stays, and no clinical response to therapy (**Figure 4**).

Subsequently, ROC analysis was performed to confirm the ability of CASE score in forecasting prognosis and calculate the optimal cutoffs. The AUC of CASE score on admission for short-term bad outcome was 0.79 (95%CI: 0.71–0.87, *p* < 0.001) (**Figure 3B**), while CASE score on admission and discharge for 1-year poor outcome was 0.78 (95%CI: 0.70–0.85, *p* < 0.001) (**Figure 3C**) and 0.89 (95%CI: 0.83–0.94, *p* < 0.001) (**Figure 3D**), respectively. According to the analysis of the ROC curve, the optimal cutoff value of CASE on admission in dichotomizing short-term and 1-year functional status was both 22.5 (sensitivity, 66.7%, specificity, 78.9% for short-term; sensitivity, 77.9%, specificity, 68.1% for 1-year). The optimal cutoff value of CASE at discharge to predict 1-year poor functional status was 9.5 (sensitivity, 80.9%; specificity, 79.2%).

### 3.7 CASE score and relapse

**Figure 5:**
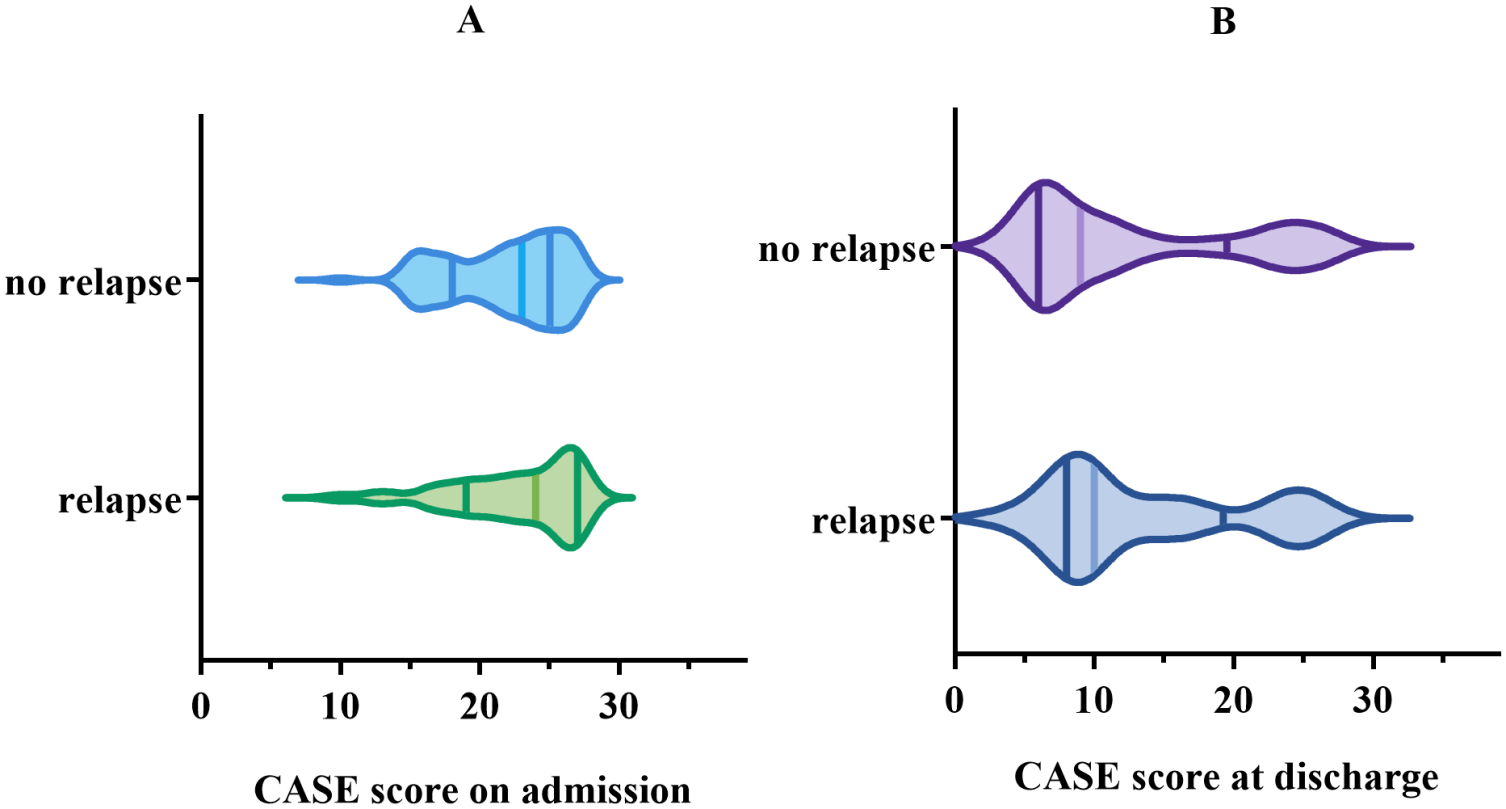
Comparison of CASE score in the group with relapse *vs*. the group with no relapse. **(A)** for CASE score on admission. **(B)** for CASE score at discharge.

At the 1-year follow-up, 43 (30.71%) patients experienced relapse, and among them, 20 patients (46.51%) experienced multiple relapses. Nonetheless, no statistically significant connection was found between CASE score and relapse, nor between mRS score and relapse (*p* > 0.05) (**Figure 5**).

## 4 Discussion

In this retrospective study, our findings are as follows: (1) CASE score presented great reliability and validity for evaluating patients with severe AE. (2) CASE score showed superiority in predicting both short-term and long-term outcomes. (3) CASE score on admission and age at onset were identified as independent risk factors for poor short-term prognosis. Factors connected with poor long-term outcomes include CASE score on admission, age at onset, duration of hospitalization, and no response to treatment. (4) Patients with CASE score > 22.5 upon admission were associated with poor treatment response and unfavorable short-term and long-term prognosis. Notably, there has been a lack of focused examination of the reliability and validity of the CASE score specifically in severe AE patients. Our research addresses this gap by validating the CASE score as an effective tool for assessing disease severity and predicting clinical outcomes in a large cohort of severe AE patients, and by establishing cut-off values for prognosis prediction.

The modified Rankin scale (mRS) is a widely used tool for evaluating the overall disability of acute stroke patients, with scores spanning from 0 to 6. Recently, this scale has been extensively utilized to evaluate the severity of AE patients. While the mRS mainly focused on the assessment of motor abilities, patients with AE frequently exhibit non-motor symptoms such as mental behavioral abnormalities, seizures, consciousness dysfunction, language disorders, etc. The CASE addresses this limitation of the mRS by providing a more comprehensive evaluation of the diverse symptoms experienced by AE patients, thus offering a more accurate reflection of disease severity. Several previous researches have demonstrated that CASE is effective in different kinds of cohorts, including the Chinese race, pediatric, and antibody positive/negative AE cohort. However, no studies have previously validated the efficacy of the CASE specifically in severe AE cohorts. Our study focused exclusively on patients with severe AE.

We found that the CASE had great item reliability in the severe AE cohort. Nonetheless, the Cronbach’s alpha coefficients for certain items contributing to the total score were low, particularly for item 1 (seizures). In the original study that developed the CASE, Cronbach’s alpha on item 1 was 0.91 (15). This discrepancy may be attributed to the fact that our cohort consisted entirely of severe AE patients. Additionally, in the validation cohort studied by Lim et al., 76.3% of patients were diagnosed with anti-NMDAR encephalitis, a condition in which seizures are a common symptom (12). In contrast, 72.8% of patients in our study were antibody-negative, and only 17.9% were diagnosed with anti-NMDAR encephalitis. The most prevalent symptom in our cohort was neuropsychiatric symptom. Furthermore, the CASE also exhibited great criterion validity compared with mRS. Consistent with previous studies (16, 17, 31, 32), a significant positive correlation was also observed between mRS score and CASE Score among patients with severe AE. In conclusion, CASE had good reliability and validity in assessing the severity of severe AE patients.

In addition to movement disorders, we found that neuropsychiatric symptoms emerged as the most frequent clinical manifestations. Cai et al. (18) and Schwarz et al. (33) also reported that mental symptoms were more prevalent in non-motor symptoms of all AE patients. This further supported that psychiatric disorders were a crucial clinical symptom of severe AE. The CASE score can reflect clinical fluctuations of non-motor symptoms, thereby facilitating accurate and timely assessment at each phase, which cannot be achieved through the mRS.

In our study, the CASE score was higher in patients receiving second-line treatment than in those only receiving first-line treatment, although no significant difference was observed. This may be due to the inclusion of patients from earlier years in our study. In previous years, second-line treatments were not widely used because of patients’ financial reasons and limitations of medical development. As a result, only 27.1% of patients received second-line immunotherapy, which differs from other studies (6). Patients with severe AE frequently experience rapid disease progression, which may seriously threaten their lives, and the cost of treatment is relatively substantial, imposing a significant financial burden on patients and their families. Therefore, early detection and effective treatment are critical for disease management and prognosis improvement.

Previous studies have shown that a higher mRS score was related to a poor response to immunotherapy (34). Our study found that the CASE score followed a similar change process as the mRS score for the same patient, and the AUC of the CASE score was 0.67. Based on ROC analysis, an optimum cutoff value of CASE as an indicator for forecasting poor response to treatment was 22.5, with a sensitivity of 79.4%, and a specificity of 53.8%. In addition, CASE scores on admission were also superior in predicting short-term prognosis and 1-year prognosis. Two optimal cutoffs were both 22.5 (sensitivity: 66.7%, specificity: 78.9% for short-term prognosis; sensitivity: 77.9%, specificity: 68.1% for 1-term prognosis). Clinicians can benefit from this finding to detect disease progression, early identify potentially critically ill patients, and promptly choose suitable treatments.

Similar to prior research, in the present study, 121 (86.4%) patients had decreased consciousness on admission, 46 (32.9%) had status epilepticus, and 60 (42.9%) required mechanical ventilation due to central hypoventilation. Notably, evaluating these diverse symptoms with mRS is inappropriate. Some patients may be in a severe neuropsychiatric state without compromised motor function (11, 31), and these patients have serious conditions but with low mRS scores. CASE can properly evaluate the numerous symptoms of patients with AE, overcome the limitations of the mRS in evaluating non-motor symptoms, and better reflect the severity of AE.

In this study, a total CASE score of 22.5 on admission was the best critical value for predicting poor prognosis. This value reflects to some extent the early sensitivity and predictive capability of CASE score in comparison to mRS score. Patients who present with multiple moderate to severe symptoms on admission face a greater risk of developing critical illness. In such instances, quantified symptoms can be regarded as an early warning signal that severe patients need to receive timely and advanced treatment. In addition, the CASE score can be used to monitor the effectiveness of treatment as well as the disease severity and progression by assessing the patient’s clinical symptoms conveniently. Therefore, it is not necessary to require all patients to review the relevant tests regularly, and these patients can be evaluated continuously during the follow-up process. The use of the CASE score allows doctors to make prompt adjustments to the treatment plan based on the CASE score, which may bring a better prognosis.

The advantages of CASE are clear, but it also has some limitations. In CASE, each item is scored 0-3, which is inadequate for evaluating some fatal symptoms, including unconsciousness and central hypoventilation. Adjusting the weighting of some items could potentially enhance CASE’s sensitivity in monitoring disease severity during the acute phase.

In the past few years, several studies have proved that the breakdown of blood-brain barrier (BBB) plays an important role in inflammatory diseases affecting the central nervous system (35, 36), and Qalb can reflect the extent of BBB breakdown. Yu et al. demonstrated that patients with anti-NMDAR-positive AE who experienced BBB disruption had a worse prognosis (37). However, in this study, the median Qalb was 12.06 (3.19-70.75), and we did not find that Qalb was a risk factor for poor prognosis. This may be related to the inclusion of patients in a more severe condition in this study and the confounding effects of other factors. The NLR serves as a biomarker, possibly reflecting inflammatory conditions in certain immune-associated diseases. A retrospective study has found that NLR at admission is linked to the prognosis of AE patients (29, 34). However, Ding et al. reported no significant correlation between NLR and prognosis in an anti-GABABR encephalitis cohort (34). Our findings were consistent with them. This distinction may be attributed to the diverse cohorts included in the various studies.

Several limitations exist in our study. First, due to its single-center, retrospective nature, this study inherently carries a risk of bias. Besides, the symptom statistics are based on CASE’s detailed grading rules, which may not fully capture the complexity of symptoms such as psychiatric disorders, memory dysfunction, and others. Second, the limited number of patients undergoing second-line treatment in our cohort may have contributed to skewed results. Furthermore, the correlation between AE antibody levels and prognosis remains unverified due to the substantial inclusion of antibody-negative individuals and the inadequacy of data on AE antibody titers in both CSF and serum. To verify our findings, a prospective, multicenter study with a larger sample size is required in the future.

## 5 Conclusion

In conclusion, the CASE has demonstrated great reliability and validity in assessing severe AE patients. This scale offers an effective means of assessing both the severity and progression of severe AE. Notably, it outperforms the modified Rankin Scale (mRS) in evaluating non-motor symptoms, demonstrating greater sensitivity in this regard. Furthermore, the CASE can be utilized to predict responses to immunotherapy and overall prognosis, which is invaluable for clinically stratifying patients and determining the most appropriate treatment strategies. This underscores the CASE’s potential as a superior tool for managing and identifying severe AE patients in clinical practice.

## Data Availability

The raw data supporting the conclusions of this article will be made available by the authors, without undue reservation.
